# Rare earth elements affect the growth and fitness of free-floating plant *Lemna minor* L.

**DOI:** 10.3389/fpls.2025.1540266

**Published:** 2025-02-28

**Authors:** Isidora Gjata, Franca Tommasi, Silvana De Leonardis, Costantino Paciolla

**Affiliations:** Department of Biosciences, Biotechnology and Environment, Università degli Studi di Bari Aldo Moro, Bari, Italy

**Keywords:** antioxidant systems, duckweed, hormesis, oxidative stress, rare earth element, toxicity

## Abstract

Rare earth elements (REEs) are increasingly utilized in modern technologies but are now recognized as emerging pollutants, with limited understanding of their impact on aquatic ecosystems. In this study, the effects of selected REEs (Ce, Nd, Gd, Dy, Yb, Ho, and Lu) in chloride form were evaluated on *Lemna minor* L., a plant species widely used as tool for the biomonitoring of the aquatic environments. Under controlled laboratory conditions, growth parameters, pigment content, oxidative stress markers, total antioxidant capacity, and antioxidant enzyme activities were assessed at millimolar concentrations over different exposure periods. *L. minor* exhibited tolerance to low millimolar concentrations of REEs over short-term exposure. However, prolonged exposure to high concentrations resulted in toxicity, characterized by growth inhibition, chlorophyll degradation, increased lipid peroxidation, and oxidative stress. Particularly, a hormetic response was observed for cerium, with stimulation at low concentrations and inhibition at higher levels, while dysprosium did not significantly affect growth. Other tested REEs induced varying degrees of stress, with holmium and lutetium causing the most severe toxic effects. Changes in antioxidant enzyme activities indicated a differential activation of stress responses depending on the REE type. These findings highlight the necessity for continuous monitoring of REEs in aquatic systems and support the use of *L. minor* as a valuable tool for environmental risk assessment.

## 1 Introduction

Rare earth elements (REEs) include elements from lanthanum to lutetium that are vital for environmentally friendly energy production, electronics, and military applications (Reisman et al., 2013). Currently, REEs are globally produced at a rate of 124,000 tons per year (U.S. Geological Survey, 2016), two orders of magnitude lower than copper or aluminum (Schüler et al., 2011). However, the production of industrially relevant REEs is anticipated to increase rapidly in the coming decades (Alonso et al., 2012). Coupled with virtually no recycling of REEs (Binnemans et al., 2013) and the difficulty in replacing REEs due to their unique properties (UNCTAD, 2014), their environmental impact is unavoidable and accelerating. The European Commission considers REEs the most problematic group of raw materials, with the highest supply risk (European Commission, 2010).

With China’s decreasing exports of REEs, all other countries in the world are facing a supply risk, and, to address the problem of supply of these rare earth elements, mining companies have been active in the search for new exploitable REE deposits and some old mines have been reopened (Humphries, 2010).

Cerium (Ce) is one of the most abundant elements among REEs, displaying a wide range of human applications. Chloride and nitrate forms of Ce and lanthanum (La) are the main constituents of REE micro-fertilizers used in China since the 1970s to improve crop yield (Hu et al., 2004). In addition, the utilization of REEs in agriculture, animal husbandry, and medicine is well known (Tommasi and d’Aquino, 2017; d’Aquino and Tommasi, 2017). The presence of anthropogenic REEs in aquatic systems was first demonstrated with the release of gadolinium (Gd) contained in contrast agents used in magnetic resonance imaging (MRI), disturbing natural REE biogeochemical cycles (Bau and Dulski, 1996). There are limited data on other REEs, notably on neodymium (Nd), terbium (Tb), and heavy (H)REEs such as thulium (Tm) and ytterbium (Yb), which should thus be studied with priority, particularly light (L)REE that are more abundant and bioaccumulated than HREE (Blinova et al., 2020). Most studies evaluated the individual effects of REEs but not the combined effects of REE mixtures (Gonzalez et al., 2014), although different REEs occur together in the environment. Missing data on the toxicity of some REEs and their mixtures prevents the establishment of a consensus on the uniformity of different REEs (Gonzalez et al., 2014).

Although REEs have received increased attention recently, little is known about their effects on the aquatic biome and aquatic organisms. However, some data indicate that their presence is growing. The presence of anthropogenic REEs in aquatic systems leads to positive anomalies (Song et al., 2017), raising environmental concerns. Despite more studies being available nowadays, REE ecotoxicology is still poorly understood (Arienzo et al., 2022).

In surface waters, REE behaves as trivalent ions, except for Ce^3+^, which can be oxidized to Ce^4+^ and precipitate in solution (Cotton et al., 1999), or Europium Eu^3+^ which is reduced to Eu^2+,^ increasing its solubility (Weltje et al., 2002).

Lanthanum and cerium are the most studied elements in the aquatic ecosystem. As regards toxicity, some studies report that the algae *Chlorella vulgaris* and *Skeletonema costatum*, treated with various lanthanides at millimolar concentrations, show signs of toxicity (den Doore de Jong and Roman, 1965; Tai et al., 2010). In *Hyalella azteca*, ecotoxicity decreases with the increasing atomic number of light lanthanides (Borgmann et al., 2005). The cause of this can be attributed to the variable composition of the culture media used in ecotoxicology and the differences related to the different REEs used. In particular, forming insoluble precipitates with phosphate and carbonate (Barry and Meehan, 2000) in some culture media probably leads to changes in the lanthanide concentration during some tests, resulting in an underestimation of their toxicity (Borgmann et al., 2005). Recent studies show adverse effects on sea urchin *Paracentrotus lividus* embryos after treatment with Ce^4+^ and La^3+^, with decreased mitotic activity and increased mitotic aberrations afterwards. At micromolar concentrations, there were adverse effects on embryogenesis (Oral et al., 2010). Among aquatic plants, the duckweed (*Lemna minor* L) is an easy-to-culture and handle vascular plant that is relatively sensitive to different pollutants, hence suitable for ecotoxicological testing (Fenske and Raymond, 2006; Moody and Miller, 2005). It is a common, relatively simple structured freshwater plant belonging to the *Lemnoideae*. Standard guidelines to perform a growth inhibition test on *L. minor* were published by the Organization for Economic Co-operation and Development (OECD, 2006). In plants, the responses to the abiotic stress induced by toxic agents with harmful overproduction of reactive oxygen species (ROS) comprise a network of antioxidant systems to counteract their toxicity. Between them, the components of the ascorbate-glutathione cycle participate in ROS-scavenging (Paradiso et al., 2016; Hasanuzzaman et al., 2019) in which the ascorbate (ASC) is a key component (Paciolla et al., 2019). Recently it has been reported that Ce affect growth and metabolism of duckweed plants following a biphasic trend, with stimulatory effects at lower concentrations and inhibitory effects at higher concentrations. Greater Ce concentrations also inducing toxicity symptoms and alteration of photosynthetic pigment content as well as alteration of ROS production, lipid peroxidation, unbalance of antioxidant systems, i.e. typical markers for stress conditions, confirming the potential biological risk associated to Ce-accumulation in soil and freshwater (Zicari et al., 2018).

This work aimed to clarify the potential toxicity of different single REEs and the differences between each one of them in aquatic environments using duckweed as a free-floating macrophyte. The effect of REEs, including Ce, Nd, Gd, Dy, Yb, holmium (Ho) and lutetium (Lu) on development (growth parameters), pigment content and redox status including oxidation level, total antioxidant capacity and antioxidant systems, were investigated under controlled conditions up to millimolar concentrations, considered as threshold levels for polluted water sites (Zhu et al., 2012).

## 2 Materials and methods

### 2.1 Plant growth

A strain of *L. minor*, registered at the Herbarium Horti Barensis (BI) n. 41980, RDSC clone 5617, was obtained from the collection of the Botanical Garden of the University Aldo Moro in Bari (Apulia, Italy). Botanical identification was confirmed on a phenotypic basis following Pignatti et al. (2017). The plants were surface sterilized by immersion in a 0.5% (v/v) sodium hypochlorite solution for 1 minute. The plant material was then rinsed with sterile water and transferred, as a number of batches, into culture vessels containing fresh modified Hoagland medium under white light at PFFD of 90 μmol m^-2^ s^-1^ with 16 h/8 h light/dark photoperiod at 24 ± 2°C for 7 - 10 days. For each experiment, representative samples of at least 40 plants with 2 visible fronds (Zicari et al., 2018) were incubated in Petri dishes of 9 cm of diameter containing 15 mL of solutions with REE chloride at concentrations 0 (control), 0.1, and 1 mM with pH 5.5. The material was harvested after 3, 7 and 12 days. The concentrations utilized in the trials were applied on the basis of the previous studies (Zicari et al., 2018) and are in accordance with the values reported in some environmental analyses (Zhu et al., 2012).

### 2.2 Chemicals and culture media

In order to find a suitable medium maximizing *L. minor* growth and limiting REE-precipitation, the Hoagland medium was used for the stock culture only. The tests were conducted using water solutions of REE (since phosphate can precipitate with lanthanides (Firsching and Brune, 1991), putting REE in the growth media with phosphates may easily mask the actual toxicity of these elements, resulting in unrealistically low toxicity. REE stock solutions were prepared by dissolution in MilliQ water (cerium chloride (CeCl_3_•6H_2_O), neodymium chloride (NdCl_3_•6H_2_O), gadolinium chloride (GdCl_3_•6H_2_O), dysprosium chloride (DyCl_3_•6H_2_O), holmium chloride (HoCl_3_•6H_2_O), ytterbium chloride (YbCl_3_•6H_2_O), lutetium chloride (LuCl_3_•6H_2_O); all purity > 99%; Sigma Aldrich, Milan, Italy). Plants were cultivated in containers filled with modified Hoagland solution containing 252 mg L^-1^ KNO_3_; 472 mg L^-1^ Ca (NO_3_)_2_ •4H_2_O; 246 mg L^-1^ MgSO_4_•7H_2_O; 66 mg L^-1^ KH_2_PO_4_; 25.4 mg L^-1^ micronutrients; pH 5.5 and semi-closed with a square Petri dish.

### 2.3 Plant growth rate

Plant growth was determined by relative growth rate (RGR) measurement, a parameter as suggested by the ISO/DIS 20079 (2005) protocol and calculated as follows:


$$\text{RGR}=(\ln N_{t}-\;\ln N_{0})/\;t$$

in which *N_0_* is the number of fronds at the beginning of the experiment, *N_t_* is the number of fronds at the selected exposure time and *t* is the exposure time (3, 7 and 12 days).

### 2.4 Chlorophyll and carotenoid contents

Untreated and treated fresh plant samples (1 g) were homogenized at 4°C with 80% acetone with a ratio of 1:15 (w/v) and the homogenates were centrifuged at 20,000 × *g* for 15 min. With a Beckmann DU-800 spectrophotometer, the absorbance at λ of 663.2, 646.8, and 470 nm was determined on the supernatant, respectively for chlorophyll *a* (chl *a*), chlorophyll *b*, (chl *b*) and carotenoids (*car*) as reported by Lichtenthaler (1987).

### 2.5 Measurement of oxidation level

The oxidation level of the material was monitored by measuring the end product malondialdehyde (MDA), which indicates the level of lipid peroxidation and sugar and amino acid oxidation, according to Lanubile et al. (2022). Briefly, plant material was homogenized with 0.1% trichloroacetic acid (TCA) with a ratio of 1:4 (w/v). After centrifugation (12,000 × *g* for 10 min), the supernatant was diluted 1:1 with a solution containing 20% TCA and 0.5% thiobarbituric acid (TBA) and incubated for 30 min at 90°C. The reaction was stopped in ice and the samples centrifuged at 12,000 × *g* for 10 min. The resulting supernatant was used for the determination of MDA-TBA complex by spectrophotometric measurement at 532 nm (extinction coefficient 155 mM^−1^ cm^−1^). The obtained absorbance was corrected subtracting the value of unspecific turbidity at 600 nm.

### 2.6 Total antioxidant capacity

ABTS (2,2′-azino-bis-(3-ethylbenzothiazoline-6-sulfonic) acid) radical-scavenging activity of the hydrophilic fractions was determined by a procedure reported by Teow et al. (2007). Plant samples (1 g) were homogenized with 85% ethanol with a ratio of 1:6 (w/v) and the homogenates were centrifuged at 20,000 × *g* for 15 min. The total antioxidant activity was determined using ABTS as a radical reacting with the different antioxidant molecules. The ABTS absorbance at λ 730 nm was determined after 1 min reaction.

### 2.7 Ascorbate and dehydroascorbate content

Treated and control plants (0.5 g) were homogenized with 5% metaphosphoric acid in a 1:4 ratio in a porcelain mortar. The homogenate was centrifuged for 15 min at 20,000 × *g* (4°C) and the supernatant was collected and immediately assayed for ASC and dehydroascorbate (DHA), according to Zhang and Kirkham (1996).

### 2.8 Total phenolic content

Samples (0.6 g) were homogenized with 5 mL of ethanol and vortexed for 1 min. The mixture was centrifuged at 6,000 × *g* for 10 min at 4 °C; 50 μL of the supernatant was added to 950 μL of distilled water and 50 μL of a 1:1 water diluted Folin-Ciocalteu reagent (Sigma Aldrich, Milan, Italy). After 3 min, 100 μL of 0.1 M NaOH solution containing 20% (w/v) Na_2_CO_3_ was added, and the resulting solution was incubated at 25 °C for 90 min (Hasperué et al., 2016). The total phenolic content was determined spectrophotometrically at 760 nm using as standard gallic acid (GA) and the results were expressed as GA equivalents (μEq GA) for g^–1^ fresh weight (FW).

### 2.9 Enzymatic activity

Samples (0.5 g) were ground with four volumes of 50 mM Tris–HCl buffer, pH 8.0, containing 0.3 M mannitol, 1 mM EDTA, and 0.05% (w/v) cysteine. The homogenate was centrifuged at 20,000 × g for 20 min at 4 °C. The supernatant was used for spectrophotometric analysis of the total proteins and enzymatic activities. The total protein content of samples was measured with bovine serum albumin as standard, according to Bradford (1976). The enzymatic spectrophotometric assays of ascorbate peroxidase (APX), catalase (CAT) and total peroxidases (POD) activities were performed according to the method described by Paciolla et al. (2008), with slight modifications. APX activity was carried out following the H_2_O_2_-dependent oxidation of ASC at 265 nm in a reaction mixture containing 50 μg of total proteins, 50 μM ASC, 90 μM H_2_O_2_ and 50 mM phosphate buffer, pH 6.5. The non-enzymatic H_2_O_2_-dependent oxidation of ASC was subtracted. CAT activity assay was evaluated by following H_2_O_2_ dismutation at 240 nm in a reaction mixture containing 50 μg of total proteins, 0.1 M phosphate buffer, pH 7.0, and 0.88 μM H_2_O_2_. POD activity was measured using 4-methoxy-1-naphthol (4-MN) as substrate. The reaction mixture contained 50 μg of total proteins, 0.1 M Tris Acetate buffer, pH 5.0, 0.1 M 4-MN, and 10 mM H_2_O_2_ in a total volume of 1 mL. The decrease in absorbance due to the oxidation of 4-MN was measured at 593 nm.

### 2.10 Statistical analysis

The data presented are the mean of at least three different replicates of three independent experiments. All analyses were run separately for each REE evaluated. One-way ANOVA followed by Dunnett’s multiple comparisons test was performed using GraphPad Prism version 9.0 (GraphPad Software, San Diego, CA, United States). Statistical significance was accepted at p < 0.01 (*), p < 0.001 (**). A Pearson correlation test was used to determine the correlations between antioxidant capacity (AC) results, total phenolics and ascorbate content. Differences of p < 0.05 were considered significant. The Scheffé test was performed to analyze the significant differences between data (p < 0.05).

## 3 Results

### 3.1 Plant growth

**Figure 1:**
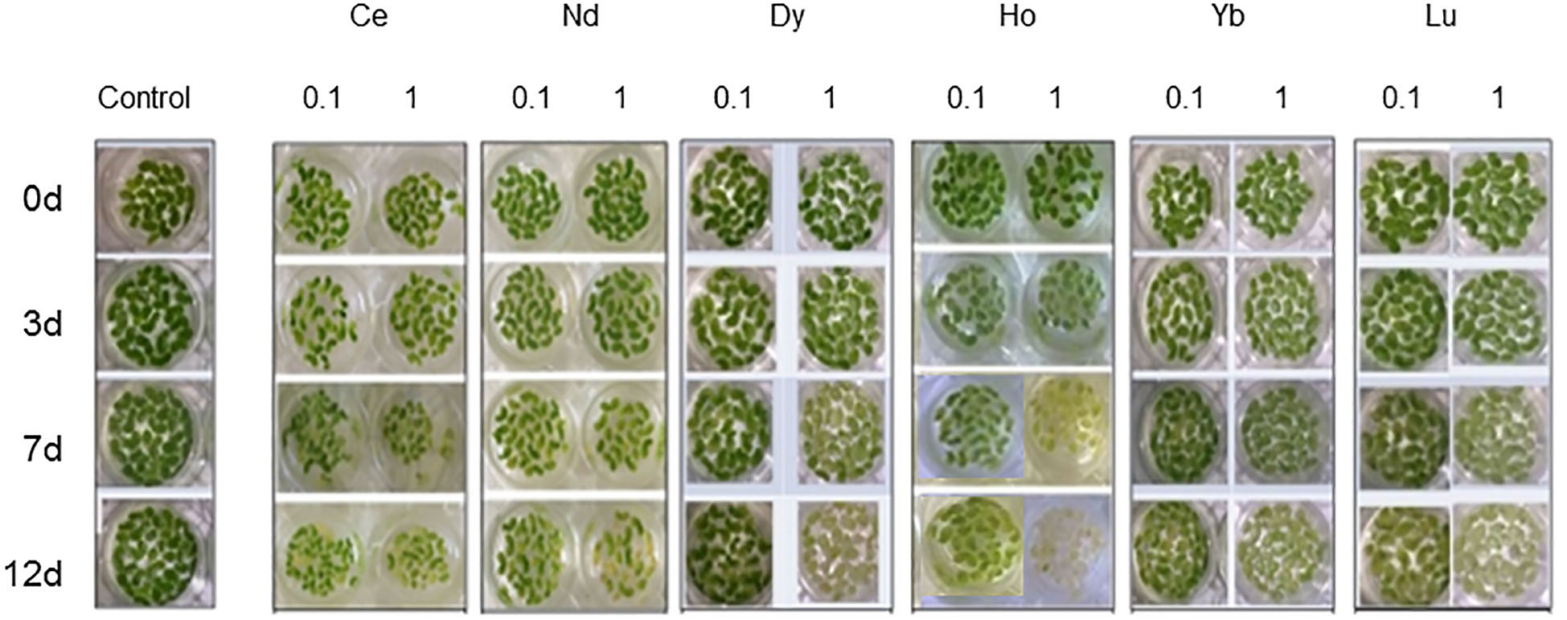
*L. minor* plants in water (control) and treated with (REE)Cl_3_•6H_2_O at concentrations 0.1 and 1 mM for 3, 7 and 12 days.

Plant growth was monitored by the RGR index (**Table 1**). A significant increase in RGR was observed only in plants treated with Ce 0.1 mM after 7 and 12 days compared to the control. A decrease was measured at 1 mM after 7 and 12 days of treatment. Nd induced a significant inhibition of the growth at 0.1 mM after 7 and 12 days of treatment. Plants treated with Gd showed a decrease in the RGR at the highest concentration after 3, 7 and 12 days. After treatment with Dy, Yb and Lu there was significant inhibition of the growth compared to the control at all concentrations except for Yb at 0.1 mM after 3 days. Plants treated with Gd showed a decrease in the RGR at the highest concentration after 3, 7 and 12 days. After treatment with Ho, a decrease in RGR was measured after 7 and 12 days of treatment for both concentrations. The appearance of marked chlorosis was observed after 7 days in plants treated with Ce, Dy, Ho, and Lu and after 12 days in plants treated with Ce, Nd, Dy and Lu at 1 mM. The plants treated with Ho at 1 mM after 12 days were in necrosis (**Figure 1**).

**Table 1:** RGR in control plants and plants treated with 0.1, and 1 mM of different REEs.

REE	RGR					
	3 days		7 days		12 days	
	0.1 mM	1mM	0.1 mM	1mM	0.1 mM	1mM
Control	6.50 ± 0.567		9.52 ± 0.8		10.05 ± 0.601	
Ce	5.26 ± 0.568	4.42 ± 0.379	14.28 ± 0.6*	3.80 ± 0.59**	15.87 ± 0.5*	3.01 ± 0.33**
Nd	6.52 ± 0.740	6.52 ± 0.201	4.85 ± 1.12*	9.52 ± 0.77	3.61 ± 0.187*	10.05 ± 0.997
Gd	7.47 ± 0.929	3.25 ± 0.60*	9.52 ± 0.297	4.18 ± 0.585*	10.05 ± 1.006	4.42 ± 0.336*
Dy	3.96 ± 0.833*	5.59 ± 0.74*	6.47 ± 0.38*	7.99 ± 1.88*	7.03 ± 0.632*	8.04 ± 0.23*
Ho	6.52 ± 1.022	6.54 ± 0.442	0.57 ± 0.2**	0.57 ± 0.11**	1.47 ± 0.21**	1.47 ± 0.12**
Yb	5.22 ± 0.576	3.57 ± 0.38*	7.23 ± 0.48*	2.28 ± 0.32**	7.63 ± 0.459*	2.51 ± 0.13**
Lu	4.22 ± 0.290*	3.57 ± 0.49*	6.28 ± 0.29*	1.90 ± 0.21**	6.33 ± 0.458*	2.11 ± 0.13**

Measurements were performed after 3, 7 and 12 days. Values represent the mean of five experiments ± SD. Statistical significance at p < 0.01 (*) and p < 0.001 (**).

### 3.2 Chlorophyll and carotenoid content

**Figure 2:**
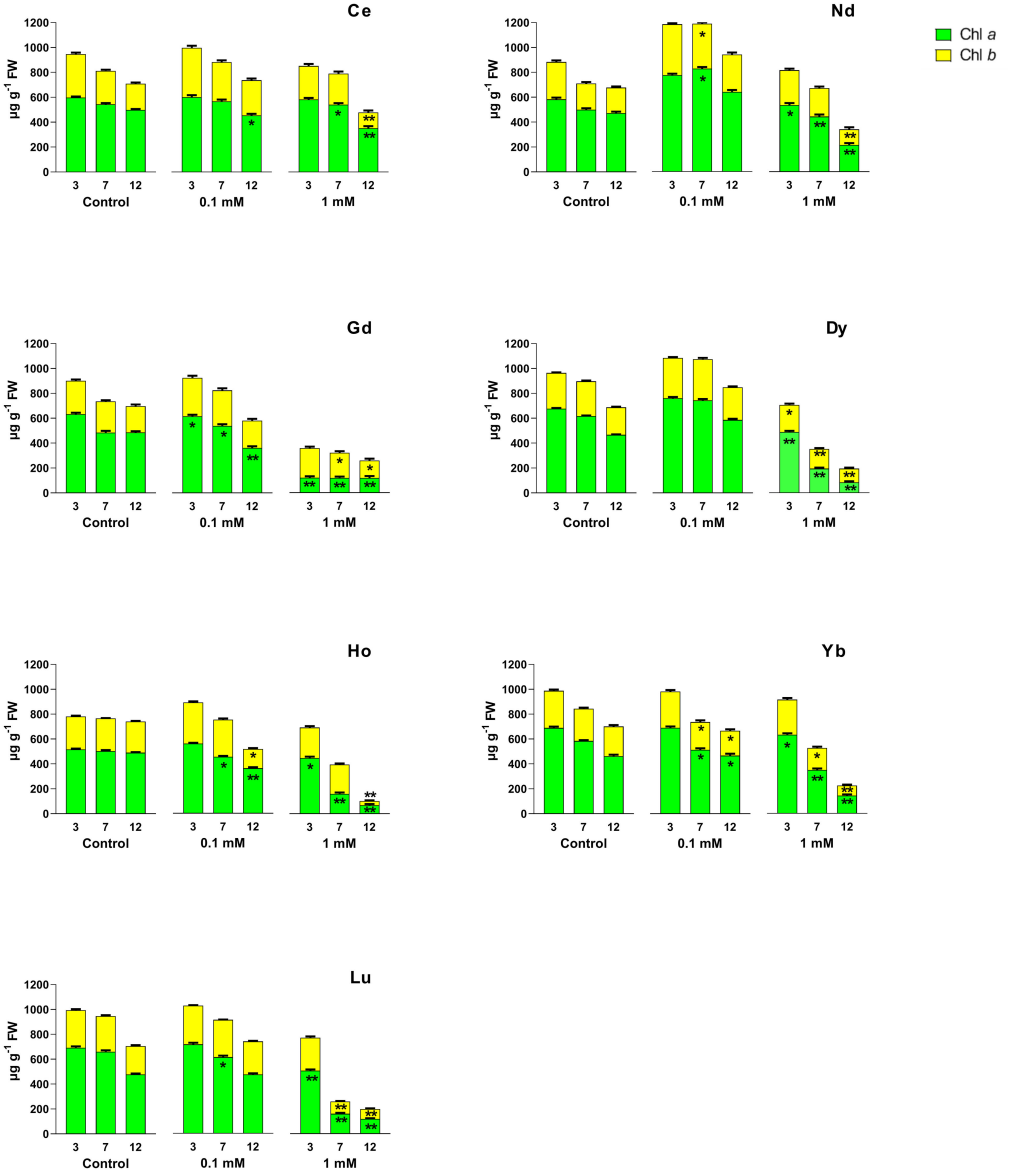
Effects of different REEs at 0 (control), 0.1 and 1 mM on the contents of chlorophyll *a* (chl *a*) and chlorophyll *b* (chl *b*) after 3, 7 and 12 days of treatment in *L. minor*. Vertical bars indicate the SD of three replicates in each treatment group. Statistical significance at p < 0.01 (*) and p < 0.001 (**); FW, fresh weight.

**Figure 3:**
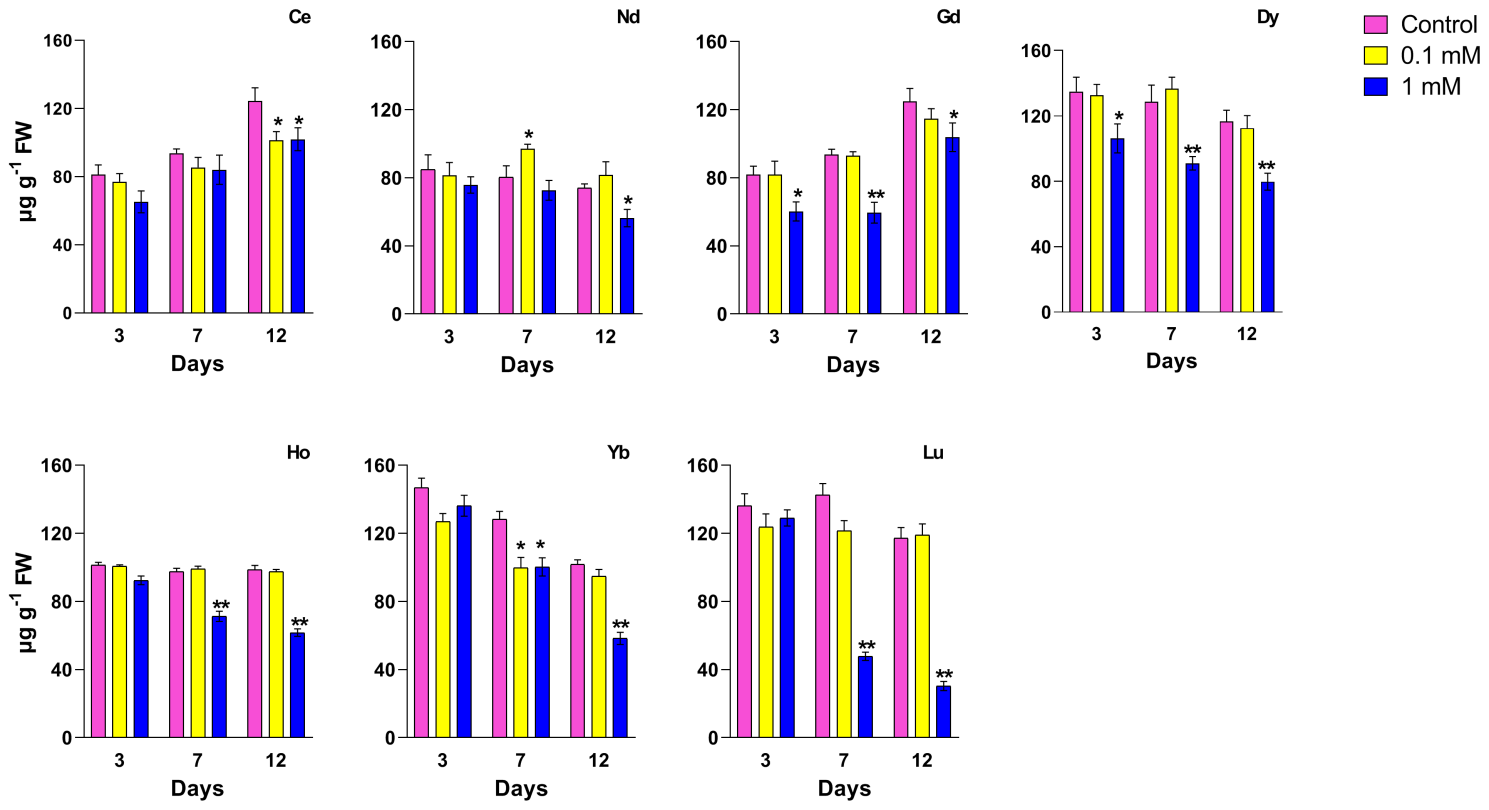
Effects of different REEs at 0 (control), 0.1 and 1 mM on the contents of carotenoids after 3, 7 and 12 days of treatment in *L. minor*. Vertical bars indicate the SD of three replicates in each treatment group. Statistical significance at p < 0.01 (*) and p < 0.001 (**); FW, fresh weight.

Chl *a* and chl *b* (**Figure 2**), and *car* contents (**Figure 3**) were affected by different REEs and concentrations. When treated with Ce, no significant changes were observed after 3 days for both concentrations. Still, after 12 days there was a significant decrease in chl *a* and *car* contents at all concentrations and after 7 days there was a significant decrease at 1 mM, compared to the control. Chl *b* content decreased significantly after 12 days at the highest concentrations. Treatments with 0.1 mM Nd did not cause significant changes in the content of photosynthetic pigments, except for day 7 of treatment, where total chlorophyll content (chl *a* + chl *b*) and carotenoids increased compared to the control. Nd 1 mM caused a significant decrease in the chl *a* content at all days of treatments; in contrast, chl *b* and *car* content decreased after 12 days. For Gd treatment, chl *a* decreased at both concentrations for the whole test duration, compared to the control, with the most significant decrease after 12 days at 0.1 mM and 1 mM. For chl *b* a significant decrease was measured after 7 and 12 days and for *car* over the whole duration of the test at the highest concentration as compared to the control. After treatment with Dy, the chl *a* and chl *b* contents decreased significantly at 1 mM compared to the control for the whole duration of the test (**Figure 2**); the same trend was observed for the carotenoid’s content when the seedlings were treated with 1 mM (**Figure 3**). Similar to 1mM Dy, a decrease at all time points was recorded in plants treated with Ho for chl *a* for both concentrations, except after 3 days at 0.1 mM, while for carotenoids, a decrease was measured at 7 and 12 days at the highest concentration. Chl *b* content was significantly lower after 12 days for both concentrations. As regards Yb, it induced a significant decrease in both chl content at 7 and 12 days for 0.1 mM and during all days of treatment for 1 mM except for chl *b* at 3 days. Carotenoid content decreased significantly after treatment for 7 days with 0.1 mM and after both 7 and 12 days of treatment with 1 mM. Chl *a* content was significantly lower than the control in plants treated with Lu at 1 mM for the whole test duration and after 7 days at 0.1 mM; for chl *b* and carotenoids a significant decrease was recorded in plants treated at the highest concentration after 7 and 12 days.

### 3.3 Ascorbate and dehydroascorbate content

**Figure 4:**
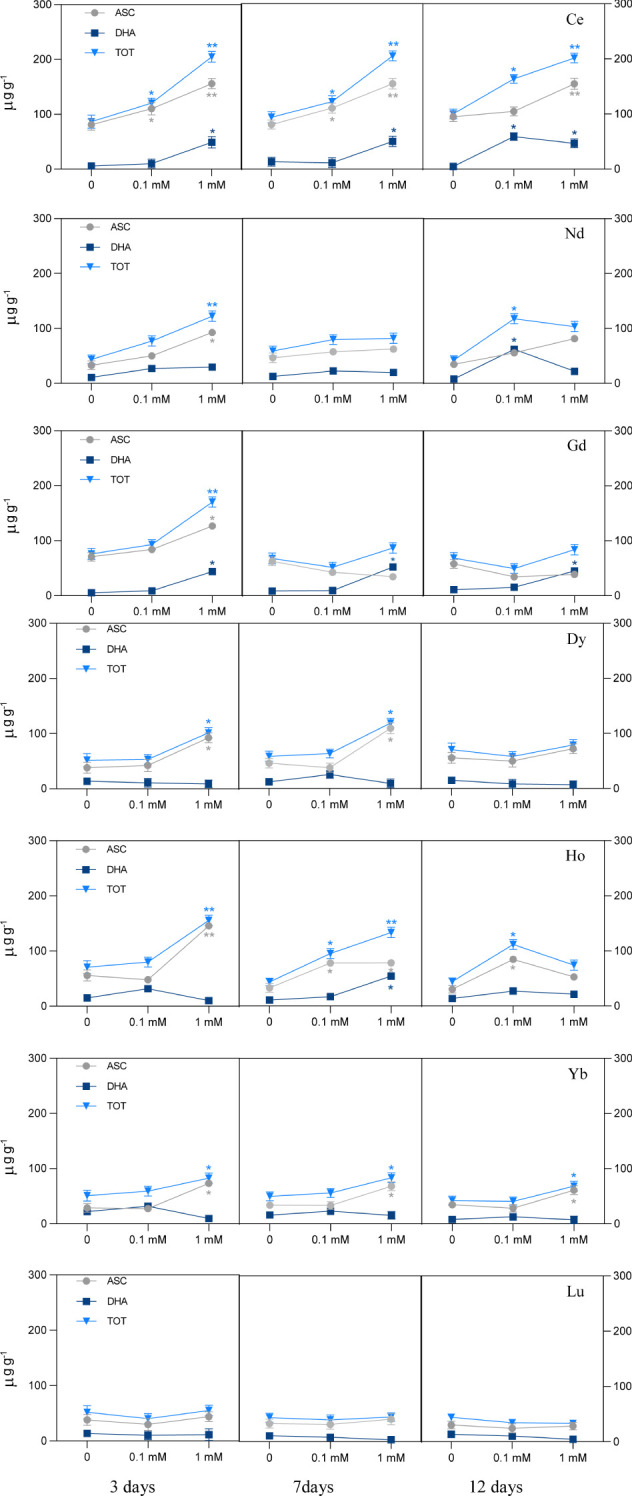
Ascorbate pool (ASC, ascorbate; DHA, dehydroascorbate; TOT, ascorbate plus dehydroascorbate) in *L. minor* treated with different REEs at 0.1 and 1 mM after 3, 7 and 12 days. Statistical significance at p < 0.01 (*) and p < 0.001 (**); FW, fresh weight.

The effect of different REEs on the ascorbate and dehydroascorbate level is reported in **Figure 4**. Exposure to Ce at 0.1 mM increased the ASC and total ascorbate (ASC+DHA), with the increase being significant after 3 and 7 days of treatment. At 12 days a significant increase was measured for DHA and ASC+DHA. When treated with the highest concentration, ASC, DHA and total ASC+DHA increased significantly in all days of exposure. Plants treated with Nd had a significant increase of ASC+DHA when treated with 0.1 mM after 12 days, due to DHA increase; a significant increase for the ASC content was measured when the seedlings were treated with 1 mM at 3 days, and as a consequence of that, of the ASC+DHA also. Exposure to Gd at 1 mM showed an increase of both ASC and DHA after 3 days whereas for the other days, DHA increased at the highest concentration, but it did not induce a significant change in the total ASC+DHA. Treatment with Dy at 1 mM showed a significant increase in ascorbate and total ASC+DHA levels after 3 and 7 days. After treatment with Ho at 0.1 mM, there was a significant increase of ASC and ASC+DHA after 7 and 12 days of treatment, while the highest concentration induced a significant increment of ASC and ASC+DHA after 3 days and of ASC, DHA and consequently total ASC+DHA after 7 days. A significant increase was shown for ASC content for plants treated with Yb at the highest concentrations for all days of treatment. No significant changes in ASC and DHA levels were measured in plants treated with Lu for the whole duration of the test, for both concentrations.

### 3.4 Total antioxidant activity

**Figure 5:**
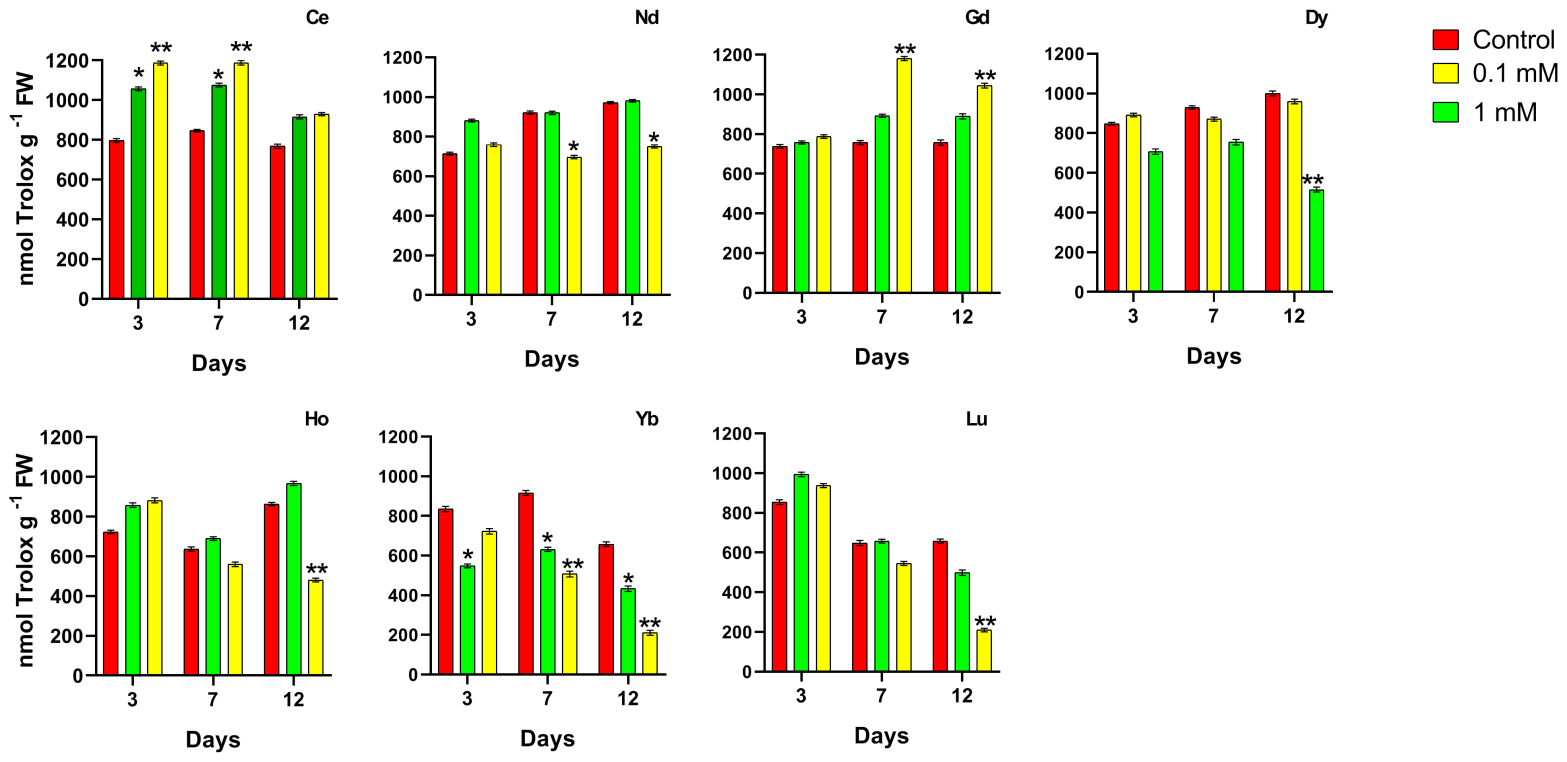
Effects of different concentrations of different REEs on the total antioxidant content after 3, 7 and 12 days of treatment in *Lemna minor*. Vertical bars indicate the SD of three replicates in each treatment group. Statistical significance at p < 0.01 (*) and p < 0.001 (**); FW, fresh weight.

The analysis of total antioxidants, which enables our work to evaluate the activity of both hydrophilic and lipophilic antioxidants as a whole, is reported in **Figure 5**.

When treated with Ce, the total antioxidant content increased with respect to the control; the plants showed a significant increase in total antioxidant level which is concentration-dependent after 3 and 7 days, while at 12 days, no significant changes were measured. Plants treated with 0.1 mM Nd showed no significant difference in antioxidant levels from the respective controls, whereas at the highest concentration this parameter was significantly lower at days 7 and 12 (about -25%). Plants treated with the highest Gd concentration showed a significant increase in ABTS after 7 and 12 days. Treatment with Dy at 0.1 and 1 mM does not induce significant changes in the total antioxidant content except at 12 days where a decrease for the highest concentration was measured. As regards to the treatment with Ho, low concentration does not cause variations in the levels of total antioxidants; in contrast, a significant decrease was observed with the 1 mM after 12 days of treatment, if compared to the control. In plants treated with Yb at 0.1 mM, a significant decrease in total antioxidants at all time points compared to the relative controls was observed; when treated with 1 mM at day 7 a decrease of about 44% reaching 68% at day 12 compared to the relative control. In plants treated with Lu, a significant decreasing effect was measured after 12 days of treatment at the highest concentration with no significant difference at other time points and lower concentration respect to the control.

### 3.5 Oxidation level monitoring

**Figure 6:**
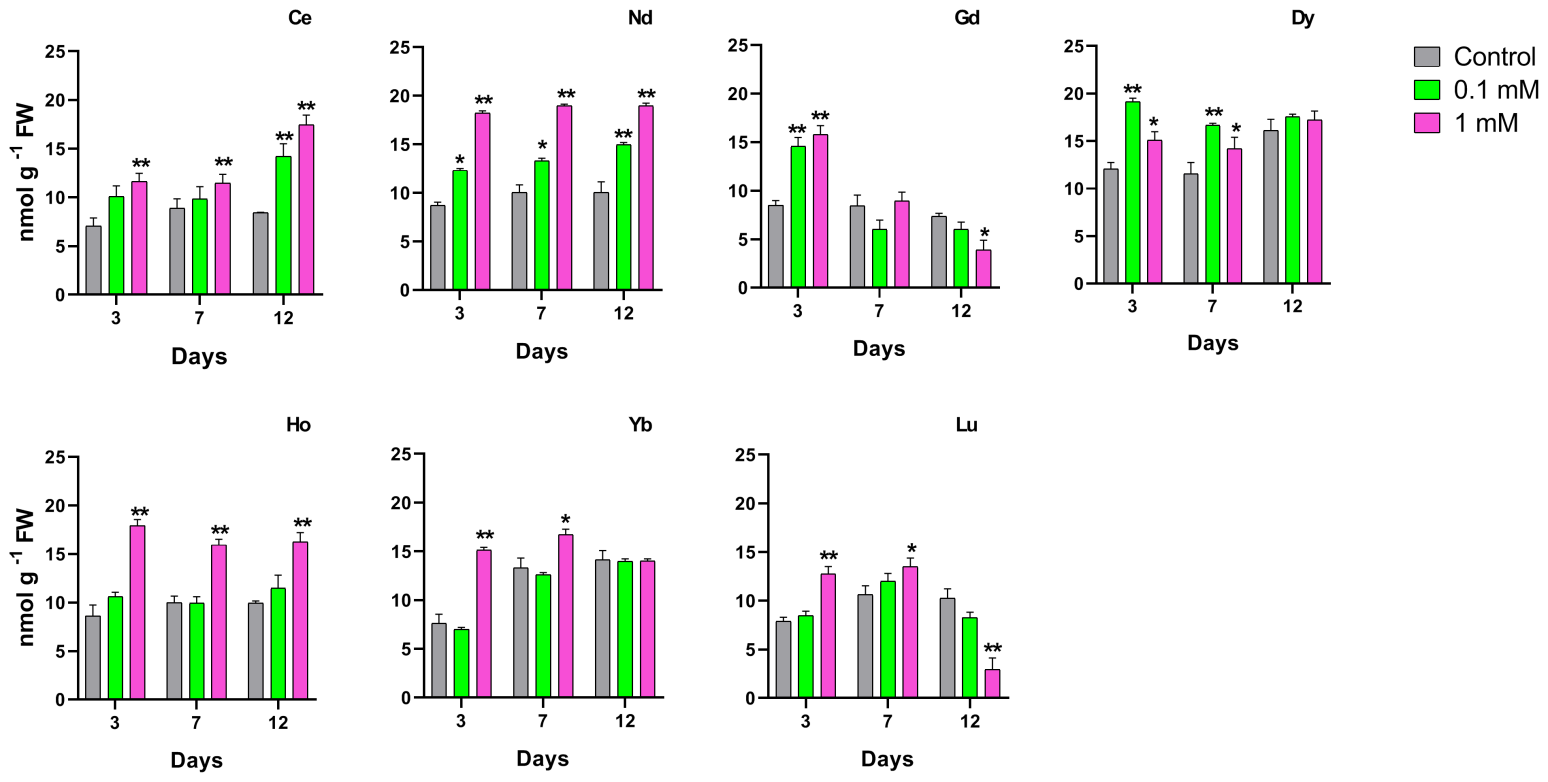
Effects of the concentrations for different REEs on the MDA content after 3, 7 and 12 days of treatment in *Lemna minor*. Vertical bars indicate the SD of three replicates in each treatment group. Statistical significance at p < 0.01 (*), p < 0.001 (**). FW, fresh weight.

The measure of the end product malondialdehyde (MDA), which indicates the level of lipid peroxidation of biological membranes, and sugar and amino acid oxidation significantly increased in plants treated with Ce, Nd and Ho at the highest concentration after 3, 7 and 12 days and when treated with Ce at 0.1 mM after 12 days and Nd at 0.1 mM for the whole duration of the test **Figure 6**. In plants treated with Gd, MDA content significantly increased for both concentrations after 3 days, whereas it significantly decreased after 12 days at the highest concentration. In Dy treatments, an increase was induced at both treatments after 3 and 7 days, while after 12 days they were not significantly different. After being treated with Ho at the highest concentration, the duckweed plant showed a significant increase in the MDA content at all time points. Treatment with Yb induced an increase after 3 and 7 days for the highest concentration; no significant changes were measured after 12 days for both concentrations. Treatments with Lu at 1 mM induced a significant increase after 3 and 7 days, while after 12 days the MDA levels were significantly lower if compared to the control.

### 3.6 Changes in total phenolic content

**Figure 7:**
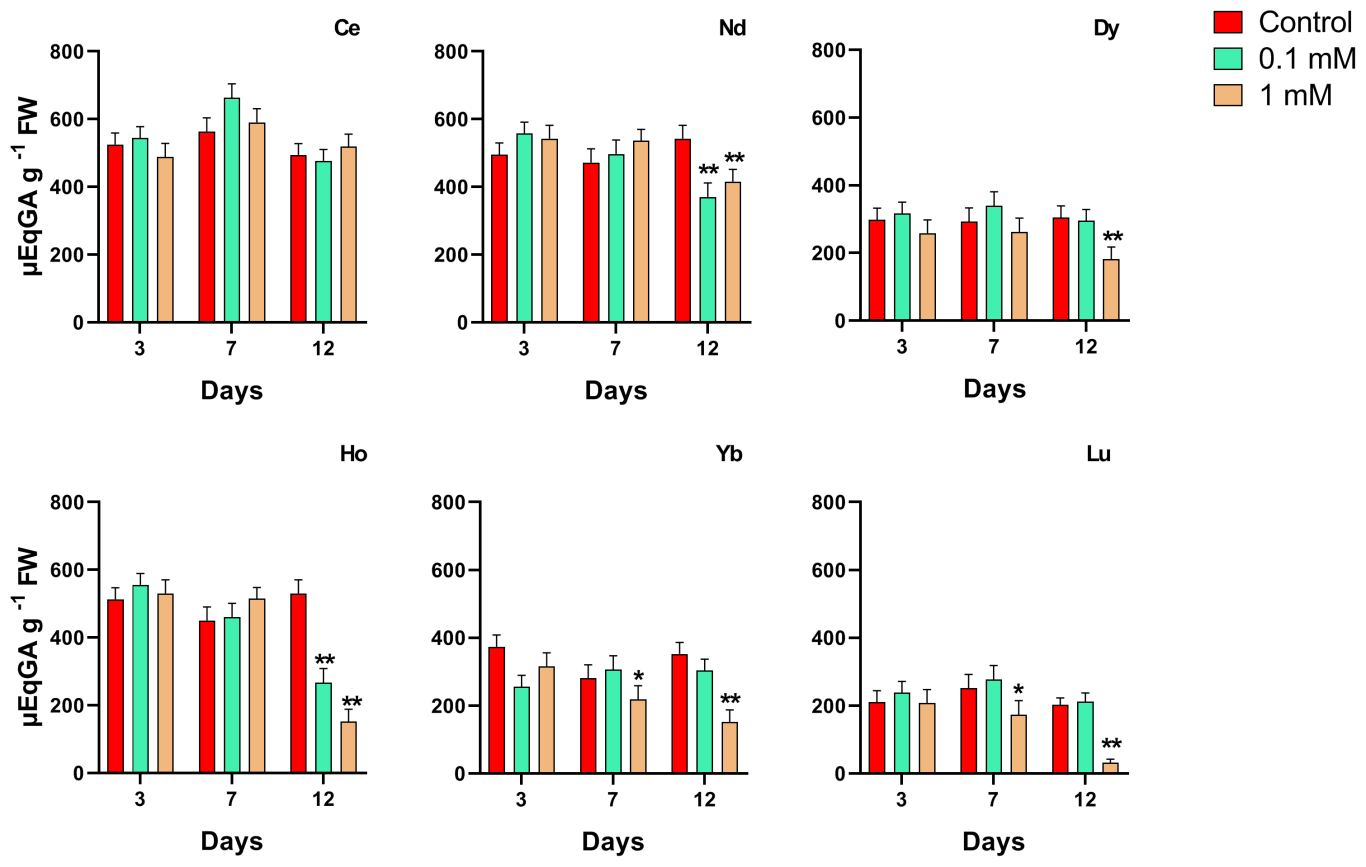
Effects of the concentrations for different REEs on the phenolic content after 3, 7 and 12 days of treatment in *Lemna minor*. Vertical bars indicate the SD of three replicates in each treatment group. Statistical significance at p < 0.01 (*) and p < 0.001 (**); FW, fresh weight.

The effect of different REEs at different concentrations and times of treatment on the total phenolic content in duckweed is shown in **Figure 7**. The longest exposure time for Nd, Dy, Ho, Lu, Yb and Lu at the highest concentration showed a significant decrease in the phenolic content. The same result was measured on plants treated with Nd and Ho at 0.1 mM after 12 days. Plants treated with Yb and Lu showed a significant decrease in the phenolic content when treated at the 0.1 mM concentration after 7 days. When treated with Lu there is a decrease after 7 and 12 days at the highest concentration.

### 3.7 Enzymatic activity

**Figure 8:**
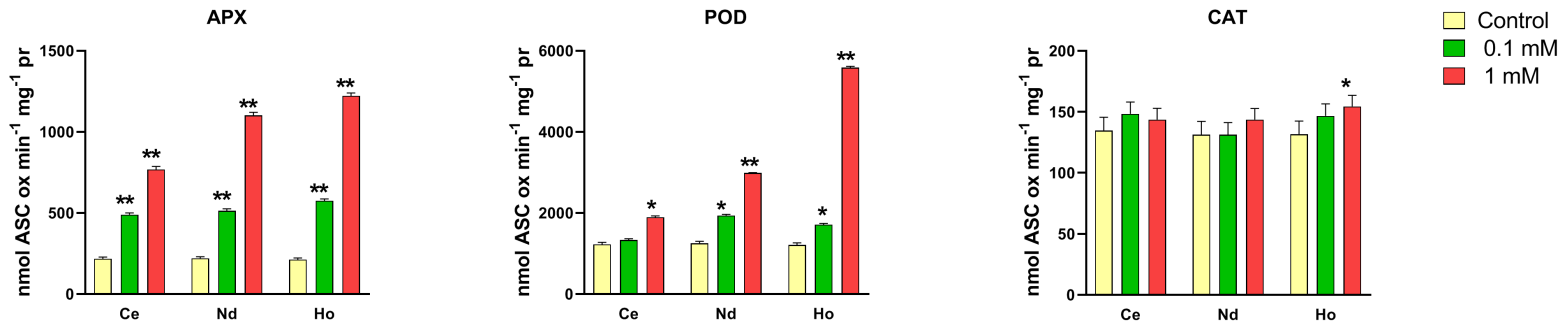
Effects of 0.1 and 1 mM of Ce, Nd and Ho on the activity of ascorbate peroxidase (APX), total peroxidase (POD) and catalase (CAT) enzymes after 7 days of treatment in *Lemna minor*. Vertical bars indicate the SD of three replicates in each treatment group. Statistical significance at p < 0.01 (*) and p < 0.001 (**); FW, fresh weight.

For this endpoint were evaluated how some of the light and heavy REEs utilized in this experimental design could affect the activity of antioxidant enzymes as CAT, APX and POD (**Figure 8**). Ce and Nd were specifically chosen due to their status as two of the most in demand and produced REEs (de Boer and Lammertsma, 2013), reflecting their ecological significance and widespread presence in environmental systems, as light REEs. Additionally, Ho was selected as a representative of heavy REEs, providing insight into the potential differential effects between light and heavy REE exposures on *Lemna minor*. The results after 7 days of treatment are shown in **Figure 8**. All treatments showed a significant increase in APX activity at all concentrations. Total peroxidase activity was different after treatment with REEs, as compared with the control. Specifically, all treatments induced a significant increase in POD activity except for Ce at the lowest concentration. Catalase activity in plants treated with Ho significantly increased at the highest concentration whereas no difference was recorded after Nd and Ce treatment.

### 3.8 Correlation between ascorbate levels and total antioxidant capacity

To define the contribution of ascorbate to the total antioxidant capacity in duckweed in the various REE treatments, the relationship between these two parameters detected was analyzed (**Table 2**).

**Table 2:** Pearson’s correlation coefficients of the antioxidant capacity and ascorbate content of *Lemna minor* after treatment for 3, 7 and 12 days with single REE.

REE	3 days	7 days	12 days
*Ce*	0.950*	0.952*	0.680*
*Nd*	0.025	-0.741*	-0.878*
*Gd*	0.986*	-0.900*	-0.729*
*Dy*	-0.955*	-0.905*	-0.947*
*Ho*	0.557*	-0.112	0.297*
*Yb*	0.152	0.372*	-0.760*
*Lu*	-0.503*	-0.999*	0.134

*Statistical significance at p < 0.01.

This test showed that there was a strong positive correlation between the two variables for plants treated with Ce that was significant during time treatments. On the other hand, when treated with Nd, Gd and Dy, ASC content was negatively correlated with the antioxidant capacity, being significant after 7 and 12 days and the latter at 3 days, too. The correlation was significantly positive for treatments with Ho after 3 and 12 days. Treatment with Yb induced a positive, medium correlation at 7 days and a negative, very high correlation at 12 days. When treated with Lu, a significant negative correlation was found at 3 and 7 days.

## 4 Discussion

### 4.1 Growth and toxicity

The increasing demand for REE in modern technology has led to a steady rise in global REE mining and extraction activities. However, little is known about these elements’ potential environmental impacts and toxicities on terrestrial and aquatic plants growing in contaminated soils (Carpenter et al., 2015) and the concentration of these elements in water and soil. *Lemna minor* is a common model organism used to study the toxicity of these elements in aquatic systems (Weltje et al., 2002). Although no negative effects were reported on *Lemna* growth at nanomolar concentrations, it is important to consider the bioaccumulation factor and monitor lanthanide emissions in water (Weltje et al., 2002). On the other hand, some data reported presence and concentration patterns of REEs in rivers in China suggesting an emerging risk of contamination (Ma et al., 2019). Data of this work confirms the potential toxicity of REEs correlated with concentration. The effects on the growth of *L. minor* plants treated with Ce chloride follow a dose-depending response inducing stimulation at 0.1 mM and inhibition at the highest concentration. This biphasic effect resembles the hormetic effect (Zicari et al., 2018) and is consistent with data reported for *Arabidopsis thaliana* (Wang et al., 2012) treated with Ce. REEs have been reported as having positive effects at low concentrations, thus suggesting a hormetic trend, implying a concentration-related shift from stimulation to inhibition and toxicity, when the concentration passes a threshold different for each species (Tommasi et al., 2023). The other REEs used in this study induced growth inhibition at both concentrations. Growth inhibition was also associated with chlorotic symptoms and, after more prolonged treatment in the case for some REEs, with necrosis, following ISO/DIS 20079 (2005) guidelines.

The accentuated leaf chlorosis under holmium treatment indicated an iron deficiency probably due to interference of this REE with the iron absorption process. Indeed, chlorosis is a characteristic symptom for plants grown in conditions of low iron levels. It manifests in decreased photosynthetic units, stromal lamellae of the chloroplast and thylakoids per granum (Spiller and Terry, 1980; Terry, 1980).

However, some REEs, such as CeCl_3_ at lower concentrations, could improve the reaction of changing yellowing seedlings into green seedlings, which suggested that Ce^3+^ may enhance the conversion of protochlorophyll into chlorophyll (Ni, 1995). While a previous study on zebrafish embryos suggested LREE to be more toxic than HREE (Lin et al., 2022), our findings show that HREEs have a much stronger and more significant effect on all endpoints measured. The same trend was reported by Alp et al. (2023), where an excess of terbium (HREE) caused toxicity and reduced plant growth in *L. minor*. On the other hand, the preferential translocation to leaves of HREEs in the five *Phytolacca* species with consequent altered growth goes along with this result (Grosjean et al., 2019).

### 4.2 Photosynthetic pigments and REEs

The observed alterations of photosynthetic pigment levels are typical markers of REEs toxicity, as reported in the responses to abiotic stress when high concentrations of them occur (Wang et al., 2014; Zhang et al., 2015). In the leaves of the aquatic plant *Hydrocharis dubia* treated with lanthanum, it is observed a decrease in pigment content, which could be related either to the disturbance in biochemical synthesis processes or to an enhanced degradation associated with damage to chloroplast ultrastructure (Xu et al., 2012). On the other hand, a number of studies revealed that several REEs may play multiple catalytic roles in chlorophyll formation and indirectly contribute to chlorophyll biosynthesis (Hong et al., 2002). A study with spinach leaves treated with concentrations of cerium at 5 and 10 µg/mL reported that Ce^3+^ increased Mg^2+^ absorption and the formation of Mg^2+^–chlorophyll, the Mg^2+^ bound to the porphyrin ring more easily than Ce^3+^, and Ce^3+^ could primarily play a catalyst role in chlorophyll formation (Hong et al., 2002). Our results align with this finding only for plants treated with Ce at the lowest concentration, where no changes in chlorophyll content were observed.

Regarding the treatment with gadolinium, a lower chlorophyll content compared to the control was observed in *Lemna* already at 0.1 mM, suggesting that this concentration was toxic for this aquatic plant. In *A. thaliana* the total chlorophyll content at low concentrations of Gd (heavy metals) (0.05 mM) appeared unaffected, while at higher concentrations (0.2 mM) it decreased significantly (Liu et al., 2021); this underlines the presence of different response between aquatic and terrestrial plants when they are treated with Gd. The effect of REEs at high concentrations on chlorophyll content can be compared to that of heavy metals. Indeed, some studies have indicated that could damage genes regulating chlorophyll in *A. thaliana*, thus altering chlorophyll content and affecting plant growth (Liu et al., 2021).

### 4.3 Effect of REEs on ascorbate content and total antioxidant capacity and their correlation

Data reported in this work show that a remarkable increase in ascorbate content was evident in duckweed plants when treated at the highest concentration throughout the whole duration of the test for all REEs, with the exceptions of Ho and Nd. Generally, increased ascorbate content highlights the plant’s attempts to counteract the oxidative stress induced by REEs (Gjata et al., 2022). It is known that ASC is a key molecule in the plant antioxidant system and due to its redox potential ranges from +0.40 to +0.50 can directly suppress the reactive species (Paciolla et al., 2019). In shorter times, there is an evident strong and positive correlation between ascorbate and antioxidant capacity, which implies an important involvement of ascorbate in the antioxidant machinery of *Lemna* after REE treatment. In this case, ASC can be considered an important antioxidant component, being sensitive to abiotic stress like REEs. Our results show however that there are also negative correlations between these two variables in plants treated with Nd, Gd, Dy and Yb after 12 days. In this case, we can assume that ASC was not directly involved or insufficient in counteracting the cellular redox imbalance due to REEs and that, after a longer time, the antioxidant machinery, including ascorbate, was compromised. On the other hand, lower ascorbate content in *Lemna* seedlings treated with Ho or Nd can be related to the fact that the plants were showing symptoms of chlorosis or were in necrosis.

### 4.4 Changes in oxidation levels

The present investigation into the effects of REEs on oxidation levels in duckweed revealed significant alterations in MDA content, an indicator for the level of lipid peroxidation as well as sugar and amino acid oxidation. The increase in MDA content at various time points indicates that the REEs induced oxidative stress in the duckweed plants, as also reported by Ippolito et al. (2010). The fact that this response varies over time suggests that the plants’ oxidative stress response is dynamic and potentially influenced by factors such as the duration of exposure and the specific properties of each REE (Gjata et al., 2022). Different REEs have distinct effects on the oxidation level. For instance, Ce and Nd consistently increased MDA levels at high concentrations at all time points, suggesting strong oxidative processes of the components in biological membrane.

In contrast, the initially increased MDA levels but later its decrease in Gd, suggested a possible adaptive response or detoxification mechanism activated by prolonged exposure. On the other hand, this trend can be in part explained by an increase in available ASC which is able to reduce tocopheroxyl radicals with a consequent decrease in lipid peroxidation (Szarka et al., 2012). Further research should explore the underlying mechanisms of these differential responses, including potential adaptive processes that may mitigate oxidative damage over longer exposure periods.

### 4.5 Changes in phenolic levels

Phenolic compounds play a crucial role in plant defense against various stresses (Lin et al., 2016). The decrease in total phenolic content suggests that the plants’ defense mechanisms are being compromised, potentially making them more susceptible to stressors (Kumar et al., 2020). The reduction in phenolic content is influenced by both the concentration of the REEs and the duration of exposure. Higher concentrations and longer exposure times generally lead to more pronounced decreases in phenolic content, indicating a cumulative negative impact on the plant’s metabolic processes over time (Wang et al., 2014). Yb and Lu show significant effects at both lower and higher concentrations, but the timing of the impact differs. This variability indicates that each REE interacts differently with the plant’s biochemical pathways. It is known that exposure to certain doses of REE induces a rise in antioxidant enzymes and in antioxidant substances such as carotenoids and total phenols of plants (Emmanuel et al., 2010a; 2010b). Such impacts of REEs do not only depend on the dose but are also associated with the duration of exposure (Zhang et al., 2013).

### 4.6 Effects of REEs on enzymatic activity

Antioxidant enzymes are important for plant defense against oxidative stress caused by ROS. These enzymes, including APX, POD, and CAT, function synergistically to detoxify ROS, are involved in maintaining redox homeostasis (Zandi and Schnug, 2022) and enhance plant stress tolerance (Hasanuzzaman et al., 2019). APX and CAT synergistically convert H_2_O_2_ into H_2_O and O_2_ (Ahmad and Tahir, 2016); particularly, APX can remove a low quantity of H_2_O_2_ more efficiently than CAT and POD, having higher affinity constant for it (μM range *vs* mM range) (Asada, 1992). Except for Ho at the highest concentration, treatments with REEs did not induce significant changes in CAT activity, suggesting that CAT was not strongly involved in breaking down H_2_O_2_, further reducing oxidative damage. Similar results have been previously detected in *L. minor* plants treated with Tb (Alp et al., 2023).

On the other hand, there was increase in activity of both APX and POD enzymes in all treatments. The increase in APX activity can also be related to the elevated levels of ASC, as when ASC biosynthesis is experimentally induced, the APX also appears earlier (De Gara et al., 1997). POD catalyzes the oxidation of various reducing agents using H_2_O_2_. Higher activity of POD could carry out increased lignification, giving strength and stiffness to plant cell walls (Habibi, 2014) and, therefore, can be considered a response to REE stressors.

### 4.7 Integrate global analysis

Data from this works suggests that REE elements have different effects on the growth and metabolism of duckweed. Cerium showed a positive effect at lower concentrations on the growth, chlorophyll content, and antioxidant systems, which was supported by no change in oxidation level. A negative effect on the growth, chlorophyll and oxidation level was observed at the highest concentration although an increase in antioxidant capacity, including ascorbate, APX and POD suggested a counteraction of *Lemna* to oxidative stress.

In the presence of neodymium, although a decrease of plant growth at lower concentrations together with higher oxidation levels of the membranes was observed, the heightened antioxidant systems appear to support the plant’s response capacity and thus lower toxicity of this REE.

In the case for gadolinium and ytterbium, the analysis of parameters studied indicated that only at the concentration of 1 mM did they assist in growth inhibition, indicating a higher tolerance of *Lemna* to these two REEs.

Dysprosium and lutetium appear to be the REEs with the highest toxicity considering the high oxidation level and low chlorophyll content with no antioxidant response.

Although a marked increase of all antioxidant enzymes analyzed was observed in the presence of holmium, this was not an efficient response to oxidative stress thus causing growth inhibition at longer times.

## 5 Conclusion

REEs are present in soil, stream water, and sewage discharge channels with different and consistent concentrations, while few data are available about the presence and mobility of REEs in these ecosystems. This study shows that *Lemna minor* can tolerate low concentrations (0.1mM) of REE chlorides, whereas toxic effects occur after treatment at the highest concentrations tested (1 mM) and/or after more prolonged exposures. Data from this work suggests that REEs differently affect the growth and induce metabolic perturbations in duckweed. The decrease in chlorophyll content and the increase in peroxidation levels are indexes of toxicity as well as the alterations of antioxidant systems although of different type and consistence depending on the REE. Cerium, neodymium, gadolinium and ytterbium appeared less toxic than dysprosium, holmium and lutetium, suggesting an increase in toxicity correlated to the molecular mass. The toxicity of REEs at millimolar concentrations observed in this study could represent a potential risk for using REE fertilizers in water cultures. The REEs are less harmful than other elements like cadmium and mercury that induce stress responses at micromolar concentrations and are also less toxic than lead, which duckweed plants tolerate at concentrations up to 0.25 mM. However, our data indicate that these rare elements represent a potential risk for aquatic ecosystems with free-floating macrophytes such as *Lemna minor*. This plant, therefore, could be a useful toxicity assessment tool in aquatic environments that may be contaminated with millimolar concentrations of REEs, although more data about the presence and mobility of REEs in these ecosystems are necessary to propose warning levels of REEs.

## Data Availability

The datasets presented in this study can be found in online repositories. The names of the repository/repositories and accession number(s) can be found in the article/Supplementary Material.
